# Quantification of FDG-PET/CT with delayed imaging in patients with newly diagnosed recurrent breast cancer

**DOI:** 10.1186/s12880-018-0254-8

**Published:** 2018-05-09

**Authors:** Christina Baun, Kirsten Falch, Oke Gerke, Jeanette Hansen, Tram Nguyen, Abass Alavi, Poul-Flemming Høilund-Carlsen, Malene G. Hildebrandt

**Affiliations:** 1 Department of Nuclear Medicine, University Hospital, Sdr. Boulevard 29, 5000 Odense C, Denmark; 2 Centre of Health Economics Research, University of Southern Denmark, Odense, Denmark; 3 University of Pennsylvania, Philadelphia, USA

**Keywords:** FDG-PET/CT, Breast cancer, Delayed-time-point, Standardised uptake values, Partial volume correction

## Abstract

**Background:**

Several studies have shown the advantage of delayed-time-point imaging with 18F-FDG-PET/CT to distinguish malignant from benign uptake. This may be relevant in cancer diseases with low metabolism, such as breast cancer. We aimed at examining the change in SUV from 1 h (1h) to 3 h (3h) time-point imaging in local and distant lesions in patients with recurrent breast cancer. Furthermore, we investigated the effect of partial volume correction in the different types of metastases, using semi-automatic quantitative software (ROVER™).

**Methods:**

One-hundred and two patients with suspected breast cancer recurrence underwent whole-body PET/CT scans 1h and 3h after FDG injection. Semi-quantitative standardised uptake values (SUVmax, SUVmean) and partial volume corrected SUVmean (cSUVmean), were estimated in malignant lesions, and as reference in healthy liver tissue. The change in quantitative measures from 1h to 3h was calculated, and SUVmean was compared to cSUVmean. Metastases were verified by biopsy.

**Results:**

Of the 102 included patients, 41 had verified recurrent disease with in median 15 lesions (range 1-70) amounting to a total of 337 malignant lesions included in the analysis. SUVmax of malignant lesions increased from 6.4 ± 3.4 [0.9-19.7] (mean ± SD, min and max) at 1h to 8.1 ± 4.4 [0.7-29.7] at 3h. SUVmax in breast, lung, lymph node and bone lesions increased significantly (*p* < 0.0001) between 1h and 3h by on average 25, 40, 33, and 27%, respectively. A similar pattern was observed with (uncorrected) SUVmean. Partial volume correction increased SUVmean significantly, by 63 and 71% at 1h and 3h imaging, respectively. The highest impact was in breast lesions at 3h, where cSUVmean increased by 87% compared to SUVmean.

**Conclusion:**

SUVs increased from 1h to 3h in malignant lesions, SUVs of distant recurrence were in general about twice as high as those of local recurrence. Partial volume correction caused significant increases in these values. However, it is questionable, if these relatively modest quantitative advances of 3h imaging are sufficient to warrant delayed imaging in this patient group.

**Trial registration:**

ClinicalTrails.gov NCT01552655. Registered 28 February 2012, partly retrospectively registered.

**Electronic supplementary material:**

The online version of this article (10.1186/s12880-018-0254-8) contains supplementary material, which is available to authorized users.

## Background

Breast cancer is the most frequent cancer among women in western countries, and up to 30% of patients are likely to develop recurrence [1, 2]. 18F-fluoro-deoxy-glucose positron emission tomography/computed tomography (FDG-PET/CT) is useful in the diagnosis, staging and therapeutic follow-up of patients with recurrent breast cancer, and is especially better than conventional imaging at detecting distant metastases [3–5].

FDG is not specific for malignancy; however, recent studies have shown the advantage of delayed or dual-time imaging with FDG-PET to distinguish malignant from benign uptake [6–8]. The underlying rationale is that malignant cells have more glucose transporters and hexokinases and less glucose-6-phosphatase (G6Pase), which leads to FDG accumulation over time compared to benign cells [9, 10]. Delayed scan time-points may thus improve the image quality due the greater difference between tumour and background levels [11–14]. This may be relevant in cancer diseases with low metabolism, such as breast cancer.

Only a few studies have examined the use of delayed time-point imaging (DTPI) in whole-body FDG-PET/CT to show the FDG accumulation over time associated with distant metastases [15–17]. The literature suggests that more prospective studies are needed to provide a better understanding of the use of DTPI in detecting recurrent breast cancer [6, 18, 19]. Analysis of PET data is often performed semi-quantitatively by measuring standardised uptake values (SUV) in lesions suspected of malignancy [20–22]. SUV has been referred to correlate well with histological and biological tumour characteristics, and can be an important tool in the diagnostic report for breast cancer patients [23–25]. SUV is strongly affected by the partial volume effect (PVE), however, which can cause a significant underestimation of the lesion uptake level [26–28]. Although methods for partial volume correction (PVC) have been developed to overcome this limitation, no method has yet found its place in daily clinical practice. Further evidence is needed to state the usefulness and feasibility of these software methods [29–32].

We aimed at examining the value of whole-body FDG-PET/CT performed at 3 h (3h) compared to the standard imaging time-point at 1 h (1h), in patients suspected of recurrent breast cancer, using quantitative software that included PVC.

Our objectives were to investigate i) the change in standardised uptake values from early (1h) to delayed (3h) time-point imaging in local and distant lesions, by measuring SUVmax, SUVmean and correcting SUVmean for PVE (cSUVmean), and ii) the effect of PVC by comparing SUVmean and cSUVmean at both time-points.

## Methods

One-hundred and two women with suspected breast cancer recurrence or with verified local recurrence and potential distant disease were enrolled in the study. The patients were part of a larger prospective accuracy study comparing FDG-PET/CT to conventional imaging in detecting recurrent breast cancer [33]. The prospective study was conducted at the PET centre, Odense University Hospital, Denmark, from December 2011 to September 2014. Exclusion criteria were history of other malignancies, age < 18 years, pregnancy or breastfeeding, diabetes mellitus, or considered unable to cooperate. For further methodological details we refer to our recent publication [33].

### FDG-pet/CT

Patients were required to fast for at least 6 h before the FDG-PET/CT scan. A maximum blood glucose level of 144 mg/dL was allowed prior to intravenous injection of 4 MBq/kg FDG. Whole-body FDG-PET/CT scans were performed 1h and 3h after FDG injection on a General Electric Discovery STE or Discovery RX system (GE Healthcare, Milwaukee, USA). A low-dose CT scan (140 kV, 30-110 mA; Auto- and Smart mA) was performed followed by a 3D PET scan. Acquisition time was of 2.5 min/frame for the 1h scan and 3.5 min/frame for the 3h scan, for patients with a normal body mass index (BMI) between 18.5 kg/m^2^ and 24.9 kg/m^2^. If BMI was lower or higher, the scan time was adjusted according to BMI and either decreased or increased by ½ min/frame, respectively. Images were reconstructed iteratively using an ordered subset expectation maximization (OSEM) algorithm, with 2 iterations, and 21 or 28 subsets, a slice thickness of 3.3 mm and matrix size of 128 × 128 (pixel size of 5.47mm^2^) with CT-based attenuation correction and 5 mm Gaussian post-filtering.

### Reference standard

Suspected recurrence was verified by biopsy. If biopsy was not possible, a composite reference standard comprising all available imaging procedures (MRI, CT, PET/CT, bone scan, ultrasound, x-ray and mammography) and/or clinical follow-up data over 6 months was used as gold standard, using the patients’ medical files as necessary. In patients with multiple lesions, it was not possible to obtain a biopsy from all lesions for ethical reasons. The patients were categorised into groups of ‘local recurrence’ or ‘distant recurrence’ based on reference standard and in accordance with treatment decision.

### Image interpretation

The scans were visually interpreted by an experienced nuclear medicine physician using the General Electric acquisition workstation. The 1h and 3h images were read independently. Each lesion was described with anatomic site and exact image number for further semi-quantitative analysis. The lesions were divided into seven subgroups according to lesion site: cerebrum, lung, liver, breast, lymph node, bone and ‘other’ (subcutaneous and muscle metastasis).

### Semi-quantitative analysis

Semi-quantitative analysis of the malignant lesions was retrospectively performed using dedicated image analysis software (ROVER™, ABX, Radeberg, Germany). This software provides semi-automatic image segmentation with a model-free method for PVE correction of SUVmean values. The software performs lesion delineation within a user-defined 3D mask using fixed, peak-based thresholding to delineate the lesion region-of-interest (ROI), which represents the metabolic active tumour volume (MTV). In the following step, ROVER performs PVC using an algorithm that defines a spill-out region of the lesion ROI from which a background corrected estimate of the spill-out region is calculated and used to perform PVC of SUVmean resulting in cSUVmean. Further details regarding software algorithms are explained by Hofheinz et al. [32, 34]. The ROVER software was used in standard mode with a threshold setting of 40% of maximum 3D mask value, including a minimum ROI volume of 1cm^3^ and excluding ROI intersection. SUV values were normalised to body weight. Manually placement of 3D masks was performed after visual identification of the lesion by the interpreting physician. Masks were placed 2-4 pixels beyond the visual margin of each lesion, and ROVER automatically delineated the lesion ROI and performed PVC. It automatically calculated ROI values of SUVmax, SUVmean, cSUVmean and MTV. Separate 3D masks were used for the same lesions in the 1h and 3h scans. If a lesion had no discernible FDG uptake in the early images, the 3D mask was placed as close as possible to the assumed origin based on anatomic orientation. A reference measurement in healthy liver tissue was obtained in all patients at both time-points. This was performed by drawing a mask of a proximately 36 cm^3^ in the upper right lobe of healthy liver tissue, avoiding malignancies and organ boundaries. Potential metastatic lesions without FDG-uptake would not be registered for analysis. The difference in SUVmax, SUVmean, cSUVmean, and MTV between the two time-points were calculated as ∆SUV=SUV3h - SUV1h, and ∆MTV = MTV3h - MTV1h.

### Statistical analysis

Descriptive statistics were performed for demographic variables and scanning parameters. The semi-quantitative analysis parameters from the 1h and 3h scans were expressed as mean ± standard deviation (SD) and range. Boxplots were used for graphical display of the data. The differences in SUVs between the two time-points as well as the difference between SUVmean and cSUVmean measurements at each time-point were estimated together with 95% confidence intervals (CI) and *p*-values that were derived from univariate linear regression models using robust standard errors to allow for intragroup correlation (i.e. multiple lesions in the same patient). Subgroup analyses were conducted by recurrence category (distant versus local recurrence) and lesion site, where healthy liver tissue was used as the reference category. Analyses were supplemented by relative changes of mean values in groups, e.g. (mean value of 3h measurements – mean value of 1h measurements)/(mean value of 1h measurements)*100%.

Statistical tests were two-sided with a significance level of 5%. Analyses were conducted with Stata/MP 14.0 (StataCorp LP, College Station, Texas 77845, USA).

## Results

Of the 102 patients who initially agreed to participate in the study one patient changed her mind before FDG-PET/CT, another was excluded due to a previous biopsy-verified bone metastasis, and a third patient did not complete the 3h scan, leaving 99 women available for analysis. Forty-one of these (41.4%) were diagnosed with recurrent breast cancer, with a total of 337 malignant lesions (mean 15, range 1-70) available for analysis. The patient and scanning characteristics are given in Table 1.

**Table 1:** Patient and scanning characteristics of 1h and 3h FDG-PET/CT, performed in the 41 patients with recurrent breast cancer

Patient characteristics	Mean ± SD, range
Patient age (years)	62 ± 4.2 [57;74]
Body mass index	27 ± 7.1 [22;32]
Blood glucose level (mmol/L)	5.4 ± 1.0 [3.8;7.7]
Years since treatment of primary breast cancer	6 ± 8.2 [0;30]
Scanning characteristics	
Dose (MBq)	281 ± 56.0 [208;401]
Time (min) between injection and early scan (1h)	62 ± 6.0 [53;80]
Time (min) between injection and late scan (3h)	180 ± 6.0 [170;200]

Nineteen patients had local recurrence comprising 21 lesions, and 22 patients had distant disease with 316 lesions. All patients had at least one biopsy to verify recurrence. Biopsies were primarily taken from breast lesions. For patients with recurrent disease and multiple distant metastases, only one distant lesion was verified by biopsy due to ethical aspect. All remaining metastatic lesions were verified by the composite reference standard, as described in the method section. Distribution of lesions and biopsies are shown in Table 2.

**Table 2:** Distribution of 337 malignant lesions in 41 recurrent breast cancer patients, according to recurrence status and lesion site. Lesion group ‘other’ consisted of subcutaneous and muscle metastases

Sites of recurrence	Number of patients	Number of lesions	Number of biopsies
Local recurrence	19	21	19
Distant recurrence	22	316	22
Cerebrum	1	1 (0.3%)	1
Lung	6	25 (7.4%)	1
Liver	4	7 (2.1%)	3
Breast	23	27 (8.0%)	21
Lymph node	13	54 (16.0%)	8
Bone	18	213 (63.2%)	7
Other	2	10 (3.0%)	5

### Local vs. distant recurrence

The overall SUV measurements for all 41 patients increased, on average, significantly between 1h and 3h, i.e., SUVmax by 1.8 (+ 28% increase), SUVmean by 1.1 (+ 28%), and cSUVmean by 2.3 (+ 35%). Overall, MTV decreased significantly over time, particularly for patients with distant recurrence in whom it decreased by 16%, shown in Table 3.

**Table 3:** Standard uptake values (SUVmax, SUVmean, partial volume corrected SUVmean (cSUVmean) and MTV of malignant lesions at 1h and 3h (mean ± SD, min and max), and the change over time (∆SUV with 95% CI) by patient recurrence status. **∆**SUV% and ∆MTV% were calculated by using mean values of 1h and 3h groups. *P*-values refer to the hypothesis test that the mean difference of the paired observations at 1h and 3h is equal to 0

	1h	3h	∆SUV (3h-1h)	p-value	∆SUV%
All 41 patients with recurrence (337 lesions)					
SUVmax	6.4 ± 3.4 [0.9-19.7]	8.1 ± 4.4 [0.7-29.7]	1.8 [1.5 to 2.1]	< 0.0001	28
SUVmean	4.0 ± 2.0 [0.5-11.3]	5.1 ± 2.6 [0.4-16.5]	1.1 [1.0 to 1.3]	< 0.0001	28
cSUVmean	6.5 ± 3.5 [0.7-20.8]	8.8 ± 4.7 [0.5-30.9]	2.3 [1.9 to 2.6]	< 0.0001	35
MTV (cc)	12.5 ± 42.4 [0.3-562]	10.4 ± 39.9 [0.2-565.5]	−2.1 [− 3.1 to − 1.0]	< 0.0001	− 17
19 patients with local recurrence (21 lesions)					
SUVmax	3.0 ± 1.9 [0.9-8.7]	3.8 ± 3.0 [0.7-12.3]	0.8 [0.2 to 1.4]	0.006	27
SUVmean	1.9 ± 1.3 [0.5-5.9]	2.4 ± 2.0 [0.4-8.6]	0.5 [0.08 to 0.9]	0.022	25
cSUVmean	3.2 ± 2.4 [0.7-8.9]	4.4 ± 3.9 [0.5-14.4]	1.2 [0.3 to 2.1]	0.014	36
MTV (cc)	8.4 ± 10.3 [0.3-33.3]	5.7 ± 5.5 [0.2-22.3]	−2.8 [−6.2 to 0.7]	0.11	−33
22 patients with distant recurrence (316 lesions)					
SUVmax	6.6 ± 3.3 [1.2-19.7]	8.4 ± 4.4 [1.2-29.7]	1.8 [1.5 to 2.2]	< 0.0001	28
SUVmean	4.1 ± 2.0 [0.8-11.3]	5.3 ± 2.6 [0.6-16.5]	1.2 [1.0 to 1.4]	< 0.0001	28
cSUVmean	6.7 ± 3.5 [0.8-20.8]	9.1 ± 4.6 [0.8-30.9]	2.3 [2.0 to 2.7]	< 0.0001	35
MTV (cc)	12.8 ± 43.7 [0.4-562.0]	10.7 ± 41.1 [0.2-565.5]	−2.1 [−3.2 to −0.9]	0.001	−16

The values of distant recurrence were in general about twice as high as those of local recurrence, but the relative average increase was the same for the two groups. In both groups, the relative increase in cSUVmean (35-36%) was greater than the increase in SUVmean and SUVmax (25-28%). Despite these average tendencies, some lesions showed reduced SUVmax values at 3h, i.e., 8 lesions (38%) in patients with local recurrence and 31 lesions (10%) in patients with distant recurrence.

### Changes by lesion subgroup

**Fig. 1:**
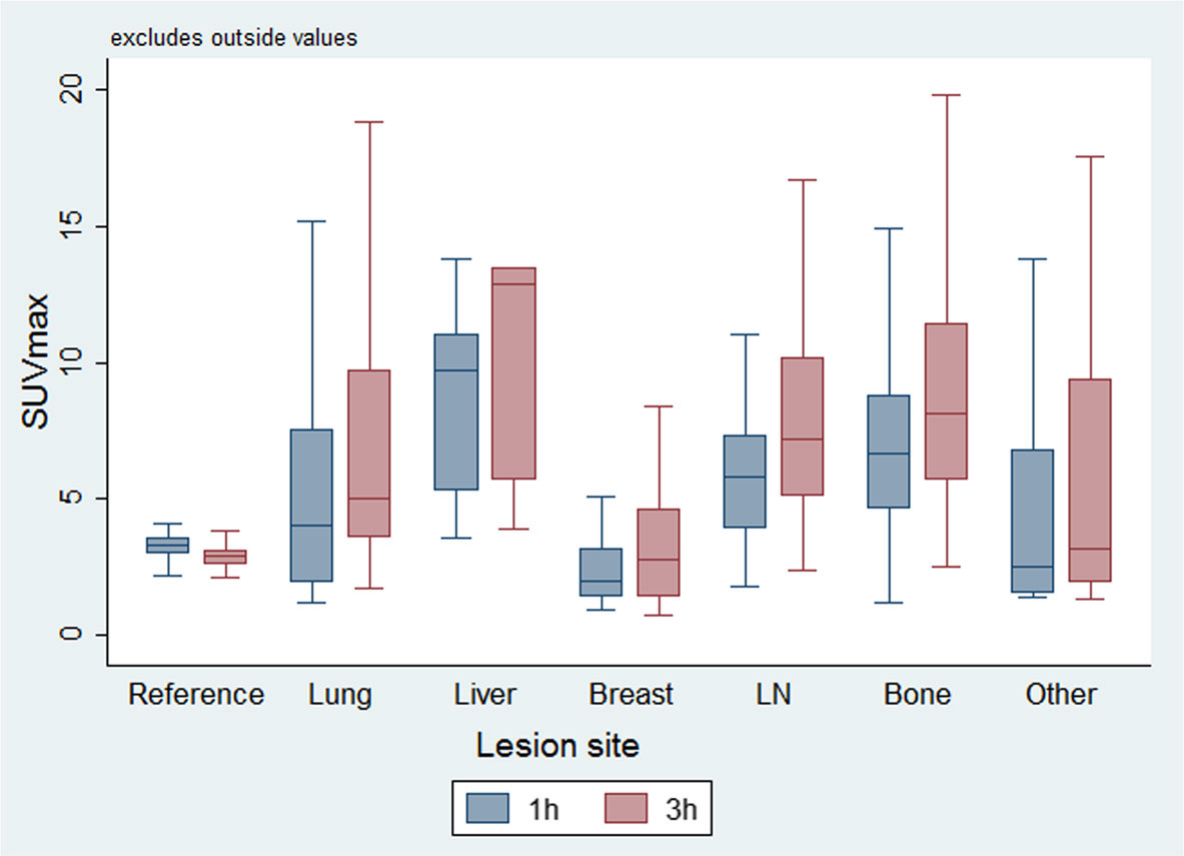
Boxplots of SUVmax, at 1h and 3h imaging time-point, for the 337 malignant lesions according to the different subgroups and reference tissue (healthy liver) in 41 patients with recurrent breast cancer. Due to only one cerebral lesion, data are not shown for this group

Except for and the lesion group ‘other’, all subgroups showed a significant increase in SUVmax and SUVmean over time, compared to the reference measurements in healthy liver tissue (Fig. 1). Lymph node metastases showed the highest absolute increase in SUVmax by 2.1 [1.4-2.8] (mean, 95% CI) (+ 33%), SUVmean by 1.5 [0.1-0.7] (+ 36%) and cSUVmean by 3.2 [2.6-3.2] (+ 47%). The highest relative increase in SUVmax, SUVmean and cSUVmean over time was seen for lung metastases at 40, 44 and 52%, respectively. Breast lesions showed the smallest absolute increase from 1h to 3h in SUVmax, SUVmean and cSUVmean at 0.7 [0.2-1.1] (+ 25%), 0.4 [0.1-0.7] (+ 24%) and 1.2 [0.4-1.9] (+ 42%). Liver lesions showed the lowest relative increase in SUVmax, SUVmean and, cSUVmean of 18, 16 and 18% respectively. Reference tissue in healthy liver showed a significant average decrease in SUVmax by 11 and 20%, respectively.

Despite the overall increase in SUV for lesion subgroups, some lesions showed reduced SUV over time, i.e., 1 (4%) in the lung, 1 (14%) in liver, 11 (41%) in breast, 3 (6%) in lymph nodes, 22 (7%) in bone and 1 (10%) of the ‘other’ lesions. The percentages of lesions with decreased values between the two time-points were the same for SUVmax, SUVmean and cSUVmean. Due to only one cerebral metastasis, this lesion group was not considered representative and is not commented upon in the results or discussion sections. MTV decreased, on average, significantly for all lesion subgroups between 1h and 3h; however, for liver lesions and ‘other’ lesions the decrease was not significant. The greatest decrease (of 43%) in MTV was seen in breast lesions. For details regarding lesion subgroups, see supplementary data given in Additional file 1.

### Partial volume correction

For all lesions as a whole, cSUVmean was significantly higher than SUVmean at both 1h (mean difference of 2.5 equal to 63%) and 3h (3.6 equal to 71%) except lesions ‘other’ and liver metastases at 1h, which was insignificantly higher (Table 4). For patients with local recurrence cSUVmean was 70% higher than SUVmean at 1h and 84% at 3h and for distant recurrence 63% higher at 1h and 71% at 3h, see Table 4.

**Table 4:** Difference between SUVmean and cSUVmean for 1h and 3h measurements for all lesions according to recurrent status and lesion subgroups (mean and 95% CI). Percentage difference was calculated by using mean values of cSUVmean and SUVmean groups for 1h and 3h. P-values refer to the hypothesis test that the mean difference of the paired observations at each time-point for SUVmean and cSUVmean is equal to 0

Group	Difference 1h (cSUVmean-SUVmean)	p-value	% diff	Difference 3h (cSUVmean-SUVmean)	p-value	% diff
All lesions (337 lesions)	2.51 [2.14 – 2.88]	< 0.0001	63	3.64 [3.22 – 4.06]	< 0.0001	71
Local recurrence (21 lesions)	1.32 [0.74 – 1.91]	< 0.0001	70	2.02 [1.07 - 2.98]	< 0.0001	84
Distant recurrence (316 lesions)	2.59 [2.21 - 2.98]	< 0.0001	63	3.75 [3.32 - 4.18]	< 0.0001	71
Lung (25 lesions)	1.88 [0.48 – 3.27]	0.018	60	3.09 [1.28 - 4.91]	0.007	68
Liver (7 lesions)	2.17 [−0.80 – 5.14]	0.103	39	2.67 [0.91 - 4.44]	0.017	41
Breast (27 lesions)	1.09 [0.59 - 1.60]	< 0.0001	63	1.86 [1.06 - 2.66]	< 0.0001	87
Lymph node (54 lesions)	2.77 [1.62 - 3.92]	< 0.0001	69	4.49 [3.16 - 5.82]	< 0.0001	82
Bone (213 lesions)	2.73 [2.43 - 2.92]	< 0.0001	63	3.80 [3.45 - 4.14]	< 0.0001	69
Other (10 lesions)	2.32 [−21.82 – 26.46]	0.437	78	2.73 [−21.54 – 27.00]	0.389	73

**Fig. 2:**
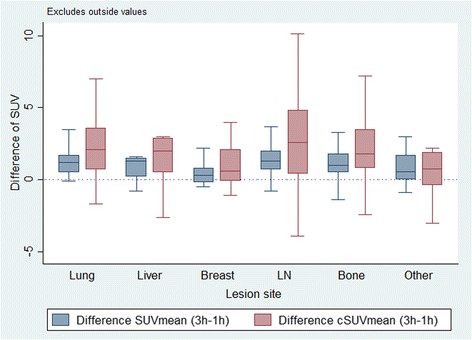
Boxplots visualising the difference from 1h to 3h in SUVmean and cSUVmean for malignant lesions, according to different subgroups and reference tissue. The difference between the two time-points were calculated as ∆SUV=SUV3h - SUV1h. Due to only one cerebral lesion, data are not shown for this group

At lesion site the largest difference was at 3h for breast lesions, where cSUVmean was 87% higher than SUVmean. The smallest difference was at 1h for liver lesions, in which cSUVmean was 39% higher than SUVmean (Table 4). Generally, cSUVmean varied more than SUVmean in all lesion sites (Fig. 2). Further details regarding lesion subgroups, see supplementary data given in Additional file 1.

## Discussion

This study of malignant lesions in 41 out of 99 analysed patients with breast cancer recurrence showed significant overall increase in uptake of FDG between 1h and 3h scans. The values of distant recurrence were in general about twice as high as those of local recurrence, but the relative increase was the same for the two groups (Table 3). Lymph node metastases showed the highest absolute increase in SUV between the two time-points, whereas lung metastases displayed the highest relative increase. PVC led to higher uptake estimates, especially for patients with local recurrence and for breast lesions at the 3h scan (Table 4 and Fig. 2). We found decreased SUV over time in reference tissue (healthy liver) as expected and hence an increased tumour-to-background ratio for delayed imaging.

Although our study, in line with previous literature, demonstrated an increased tumour-to-background ratio in delayed images, the diagnostic accuracy at patient level did not improve in our overall prospective accuracy study [33]. The clinical usefulness of delayed imaging in this category of patients may thus be limited. Furthermore, it is our experience that DTPI caused planning challenges in daily workflow and patient discomfort due to a longer fasting period.

### Change in SUV from early to late time-point imaging

Patients with local recurrence had in general lower SUVmax and SUVmean measurements compared to the group with distant recurrence at both time-points. This could partly be due to the finding that 8 of the 21 lesions (38%) in the group with local recurrence showed decreased SUV from 1h to 3h compared to only 10% in patients with distant recurrence. Suga et al. reported similar results from 52 patients with suspected local recurrence of breast cancer, where SUVmax increased in 84% of the lesions and decreased in 16% of the lesions between 1h and 2h scans, while overall SUVmax increased by 18% [35]. The higher increase in SUVmax in our study could be due to the later imaging point at 3h. Several studies have performed delayed or dual-time imaging in breast cancer but have only shown a small improvement in detecting local recurrence, despite an increased tumour-to-background ratio [11, 12, 36, 37].

Distant metastases in lung, lymph node, liver and bone all increased in SUVmax, SUVmean, and cSUVmean from 1h to 3h, and especially for lymph node metastases. These findings are supported by the literature, where several publications have stated the increased FDG accumulation over time in different malignant lesions [7, 16, 17, 35].

In our study, we used healthy liver tissue for reference measurement, which has previously been demonstrated useful by Chirindel et al. [38]. For bone metastases, we found a significant increase for SUVmax, SUVmean, and cSUVmean between 1h and 3h. These findings are supported by a study of bone metastases in breast cancer patients from Tian et al. [39]. Other diagnostic studies of bone metastases and FDG-PET/CT have found that FDG has a high sensitivity for detecting osteolytic and mixed bone lesions compared to osteoblastic lesions, which can be false-negative due to low metabolic activity [40, 41].

SUV can be influenced by a range of physiological and technical parameters, which should be taken in consideration by quantitative image analysis, to minimize bias [42–44]. SUVmax demonstrates a high inter-observer reproducibility and is often used as a semi-quantitative measure of FDG-uptake. However, SUVmax is more sensitive to image noise, and has been shown to have a lower inter-study repeatability than SUVmean [45, 46]. While SUVmean may be a more reliable measure in heterogeneous tumours, it can be observer-dependent due to lesion delineation dependency with variability in mask placement and sensitivity to PVE, especially in smaller lesions [26, 27, 47].

### Impact of PVC performed with ROVER software

PVC of SUVmean had as expected a significant impact in our study, in both the overall lesion group and the various subgroups. However, PVC increased the standard deviation of cSUVmean compared to uncorrected SUVmean, probably due to incorrect lesion delineation caused by segmentation challenges (Fig. 2). The highest impact of PVC was seen for breast lesions which also had the smallest MTV according to ROVER. Our results agree with the literature, showing that partial volume effects influence measured uptake in all lesions, especially those smaller or of a size close to the limited spatial resolution of the PET scanner, for which it causes a significant underestimation of lesion extent and activity level [26, 27, 47].

**Fig. 3:**
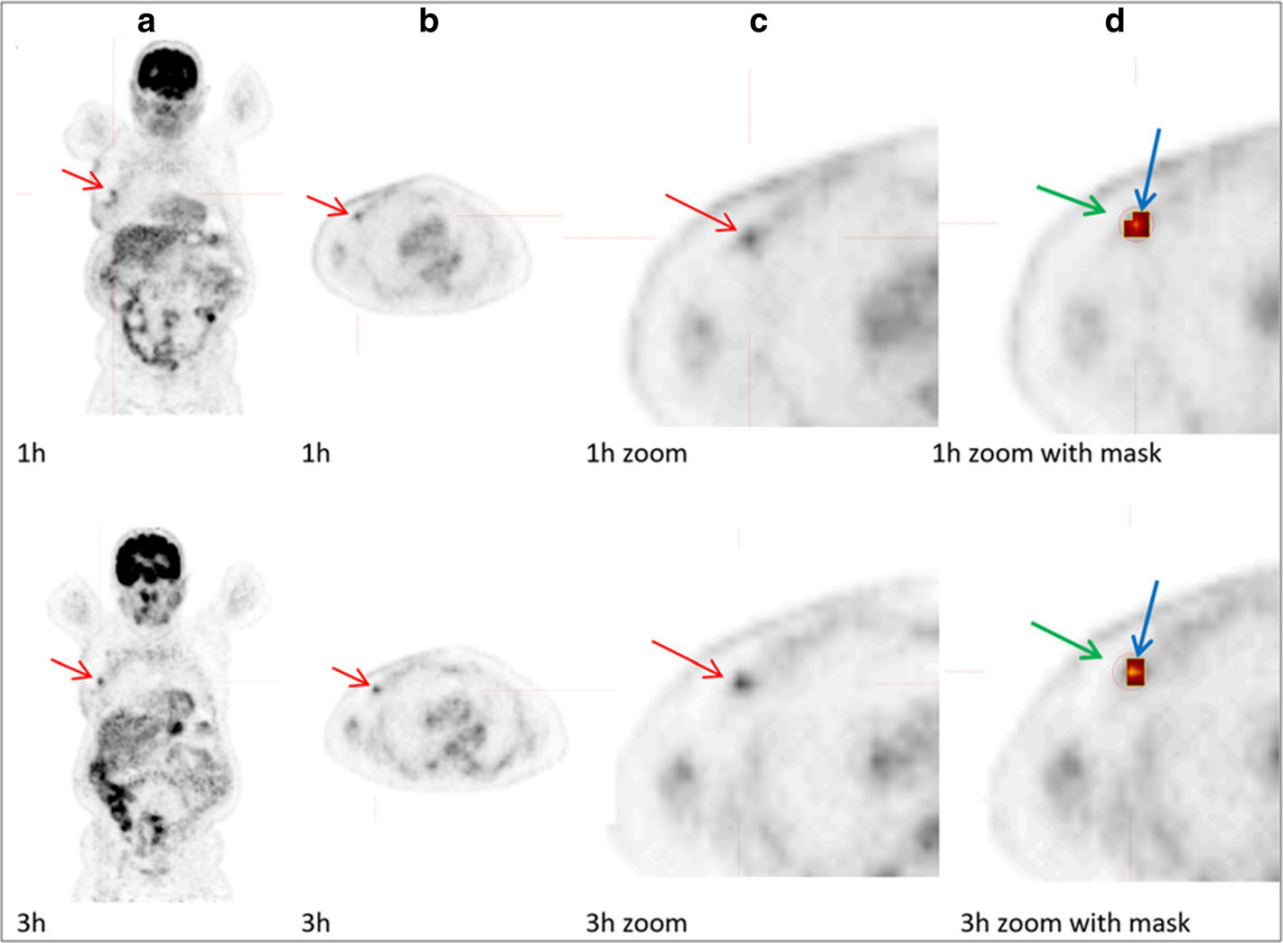
FDG-PET/CT images of a 73 years old woman with local recurrence in her right breast (red arrow) displayed in ROVER. **a** Maximum intensity projection images 1 h (1h) and 3 h (3h) after injection, **b** Transaxial images of thorax at 1h and 3h, **c** Zoomed transaxial images of the left side of thorax at 1h and 3h, and **d** Zoomed transaxial images with masks at 1h and 3h, where the green arrow shows the user defined 3D mask (equal mask size at 1h and 3h images), and the blue arrow shows the lesion ROI delineated by the ROVER software, which yielded the following values after 1h and 3h, respectively: MTV (cc) 3.8 and 2.1; SUVmax 3.2 and 3.7; SUVmean 1.8 and 2.6; cSUVmean 2.3 and 5.1

Several studies have demonstrated observer-related variability associated with manual delineation of ROI, which can be reduced by the use of automatic or semi-automatic contour drawing [48–50]. Prevalently employed automatic delineation methods employing different threshold and cut-offs, however, are also known to be suboptimal in many cases, leading to segmentation bias [20, 49]. We used ROVER software for PVC, which has previously been shown to be feasibly and clinically useful [31, 34]. ROVER software included background subtraction for each lesion, but despite this we discovered practical challenges due to non-uniformity of lesions and background activity. We experienced against expectation a decrease in MTV defined by ROVER from 1h to 3h, despite the general known tendency of increased FDG accumulation over time in malignant lesions. This issue was probably caused by crucial segmentation challenges associated with the semi-automatic lesion delineation. Thus, by visual inspection of the automatic lesion delineation in our study, the lesion ROI in the 1h image often included background voxels and thereby overestimated lesion size, compared to the same lesion in the 3h image, where the lesion delineation appeared more well-defined (Fig. 3). This indicates that the threshold-based segmentation in ROVER led to overestimated volumes of small lesions, particularly in the early scan, where the lesion-to-background ratio was low, and through that an underestimation of SUVmean. We used a fixed 40% threshold setting to semi-automatically delineate lesions. This approach was based on the current use in the literature and similar to the threshold of 41% recommended by updated European guidelines [48, 51]. A more systematic search of other threshold levels or rather alternative segmentation methods that can provide more accurate lesion delineation could be beneficial [32, 42, 49], but lies outside the scope of this article.

### Strengths and limitations of the study

The main strengths of our study are its prospective design and clinically representative patient group with newly diagnosed recurrence – verified by biopsy in all patients – and, hence, yet untreated metastases. The scanning protocol consisted of whole-body scans at both 1h and 3h allowing us to compare SUV measurements over time in all recurrent lesions. A major limitation was that histological proof was not available for all lesions due to ethical and practical reasons, and therefore a composite reference standard was the best option. Another limitation was the observed suboptimal segmentation with a fixed 40% of maximum value threshold. Segmentation methods using separate masks for 1h and 3h images in each patient were associated with challenges such as spatial mismatch between 1h and 3h acquisitions. This could contribute to segmentation variability and potentially incorrect comparison of quantitative results from the two time-points. Being the outcome of a single institution study, the generalizability of our results is uncertain.

The overall intention with this study was to consider whether a 3h scan should replace the 1h standard imaging in patients with metastatic breast cancer. Although we found an increased tumour-to-background ratio in 3h compared to 1h scans, this was not associated with improved diagnostic accuracy on a per-patient level as shown in our previous publication [33]. Furthermore, 3h protocols cause challenges regarding planning, patient discomfort and healthcare costs. Based on these experiences, it may not be justified to replace the standard 1h by a delayed imaging protocol.

## Conclusion

SUVs of FDG increased significantly from 1h to 3h in malignant lesions of recurrent breast cancer and in all types of lesions, while reference measurements in healthy liver tissue decreased. PVC increased these values significantly as expected, especially in breast metastases. However, the demonstrated modest quantitative advances of 3h imaging can hardly justify delayed PET imaging on a routine basis in this patient group.

## Additional file

Additional file 1: SUV lesion measures for various subgroups. SUV and MTV for all malignant lesion according to the different subgroups at 1h and 3h, and the change over time. (PDF 232 kb)

## Abbreviations

1h: 1 h
3h: 3 h
BMI: Body mass index
CI: Confidence interval
cSUVmean: Partial volume corrected SUVmean
CT: Computed tomography
DTPI: Delayed time-point imaging
FDG-PET/CT: ^18^F-fluoro-deoxy-glucose positron emission tomography/computed tomography G6Pase Glucose-6-phosphatase
MBq: Mega Becquerel
MRI: Magnetic resonance imaging
MTV: Metabolic active tumour volume
OSEM: Ordered subset expectation maximization
PVC: Partial volume correction
PVE: Partial volume effect
ROI: Region-of-interest
SD: Standard deviation
SUV: Standard uptake values
SUVmax: Maximum standard uptake value
SUVmean: Mean standard uptake value
