# Supplementary material for: Quantification of FDG-PET/CT with delayed imaging in patients with newly diagnosed recurrent breast cancer
Source: BMC Med Imaging. 2018 May 9;18:11. doi: 10.1186/s12880-018-0254-8 (PMC5943993; doi:10.1186/s12880-018-0254-8)
Supplement: Supplementary file 1 — SUV lesion measures for various subgroups. SUV and MTV for all malignant lesion according to the different subgroups at 1h and 3h, and the change over time. (PDF 232 kb) [file 12880_2018_254_MOESM1_ESM.pdf]

Lesion site	SUV	Time point 1h	Time point 3h	ΔSUV (3h-1h)	p- value	ΔSUV%
**Cerebrum**	SUVmax	10.7	12.5	1.8	-	17
	SUVmean	6.2	6.5	0.3	-	5
	cSUVmean	7.6	8.3	0.7	-	9
	MTV (cc)	7.6	12.5	4.9	-	65
**Lung**	SUVmax	5.1±3.5 [1.2-15.2]	7.1±4.8 [1.7-18.8]	2.0 [1.2 to 2.8]	0.001	40
	SUVmean	3.1±2.2 [0.8-8.9]	4.5±3.1 [0.9-12.9]	1.4 [0.7 to 2.1]	0.003	44
	cSUVmean	5.0±4.1 [0.8-14.2]	7.6±5.5 [0.9-19.6]	2.6 [1.1 to 4.1]	0.007	52
	MTV (cc)	5.0±4.4 [0.7-16.1]	3.7±4.0 [0.6-16.4]	-1.3 [-2.3 to -0.4]	0.014	-27
**Liver**	SUVmax	9.1±3.5 [3.6-13.8]	10.7±4.1 [3.9-13.5]	1.6 [-0.9 to 4.1]	0.14	18
	SUVmean	5.6±2.4 [2.2-9.5]	6.5±2.4 [2.4-8.7]	0.9 [-0.5 to 2.2]	0.13	16
	cSUVmean	7.8±4.5 [2.5-16.5]	9.1±3.6 [3-13.9]	1.4 [-1.4 to 4.2]	0.22	18
	MTV (cc)	192.6±225.6 [3.6-562]	174.0±225.9 [3.9-565.5]	-18.6 [-39.2 to 1.9]	0.06	-10
**Breast**	SUVmax	2.7±1.7 [0.9-8.7]	3.4±2.7 [0.7-12.3]	0.7 [0.2 to 1.1]	0.008	25
	SUVmean	1.7±1.2 [0.5-5.9]	2.1±1.9 [0.4-8.6]	0.4 [0.1 to 0.7]	0.015	24
	cSUVmean	2.8±2.3 [0.7-8.9]	4.0±3.7 [0.5-14.4]	1.2 [0.4 to 1.9]	0.004	42
	MTV (cc)	14.7±19.8 [0.3-82.9]	8.4±10.1 [0.2-42.4]	-6.3 [-11.2 to -1.3]	0.015	-43
**Lymph node**	SUVmax	6.4±3.3 [1.8-19.3]	8.4±4.8 [2.4-29.7]	2.1 [1.37 to 2.77]	<0.0001	33
	SUVmean	4.02±2.1 [1.0-11.3]	5.48±2.9 [1.3-16.5]	1.5 [0.1 to 0.7]	<0.0001	36
	cSUVmean	6.8±4.1 [1.1-20.8]	10.0±6.1 [1.5-30.9]	3.2 [2.6 to 4.1]	<0.0001	47
	MTV (cc)	4.5±5.8 [0.5-36.2]	2.9±3.6 [0.5-17.9]	-1.6 [-2.8 to -0.4]	0.015	-35

**Bone**	SUVmax	7.0±3.1 [1.2-19.7]	8.8±4.0 [2.5-21.2]	1.8 [1.4 to 2.3]	<0.0001	27
	SUVmean	4.4±1.8 [0.8-9.7]	5.5±2.3 [1.5-12.0]	1.1 [0.8 to 1.4]	<0.0001	26
	cSUVmean	7.1±3.0 [0.8-14.0]	9.3±3.9 [2.1-21.1]	2.2 [1.7 to 2.7]	<0.0001	31
	MTV (cc)	9.6±15.5 [0.4-131.5]	8.3±12.0 [0.5-89.8]	-1.3 [-70.2 to 17]	0.023	-14
**Other**	SUVmax	4.5±4.2 [1.4-13.8]	5.6±5.2 [1.3-17.6]	1.1 [-8.9 to 11.7]	0.39	25
	SUVmean	3.0±3.0 [0.8-9.3]	3.8±3.4 [0.7-11.3]	0.8 [-5.5 to 7.0]	0.36	26
	cSUVmean	5.3±5.4 [1.0-14.8]	6.5±5.7 [1.0-16.7]	1.2 [-5.2 to 7.5]	0.26	22
	MTV (cc)	4.6±8.3 [0.4-27.7]	3.8±6.1 [0.2-20.4]	-0.8 [-15.4 to 13.8]	0.62	-17
**Reference**	SUVmax	3.3±0.7 [2.2-6.1]	3.0±0.5 [2.1-4.2]	-0.4 [-0.5 to -0.2]	<0.0001	-11
	SUVmean	2.4±0.4 [1.6-4.0]	1.9±0.3 [1.4-2.6]	-0.5 [-0.6 to -0.4]	<0.0001	-20
	MTV (cc)	36.4±5.0 [6.0-38.0]	36.2±4.2 [11.1-37.3]	-0.2 [-0.7 to 0.3]	0.37	-0.8

Additional table 1, SUV lesion measures for various subgroups. Standard uptake values (SUVmax, SUVmean and partial volume corrected cSUVmean) and MTV of malignant lesions at 1h and 3h (mean ±SD, min and max), and the change over time (ΔSUV with 95% CI) according to the different lesion subgroups. ΔSUV% was calculated by using mean values of 1h and 3h groups. P-values refer to the hypothesis test that the mean difference of the paired observations at 1h and 3h is equal to 0.
